# A summary of cryptosporidiosis outbreaks reported in France and overseas departments, 2017–2020

**DOI:** 10.1016/j.fawpar.2022.e00160

**Published:** 2022-04-29

**Authors:** Damien Costa, Romy Razakandrainibe, Louise Basmaciyan, Jérôme Raibaut, Pascal Delaunay, Florent Morio, Gilles Gargala, Venceslas Villier, Abdelmounaim Mouhajir, Bernard Levy, Catherine Rieder, Sébastien Larreche, Sophie Lesthelle, Noémie Coron, Estelle Menu, Magalie Demar, Vincent Pommier de Santi, Véronique Blanc, Stéphane Valot, Frédéric Dalle, Loic Favennec

**Affiliations:** a Department of Parasitology/Mycology, University Hospital of Rouen, 76000 Rouen, France; b EA ESCAPE 7510, University of Medicine Pharmacy Rouen, 76000 Rouen, France; c CNR-LE Cryptosporidiosis, Santé Publique France, 76000 Rouen, France; d CNR-LE Cryptosporidiosis Collaborating Laboratory, Santé Publique France, 21000 Dijon, France; e Regional Health Agency PACA, Santé Publique France, 13002 Marseille, France; f Parasitology and Mycology Department, Université Côte d'Azur, CHU Nice, 06000 Nice, France; g Parasitology-Mycology Laboratory, Institut de Biologie, CHU de Nantes, 44093 Nantes, France; h INSERM: Institut National de la Santé et de la Recherche Médicale, 75015 Paris, France; i Laboratoire SCHUH, BIO67-BIOSPHERE, 67000 Strasbourg, France; j HIA Begin, 94160 Saint Mandé, France; k Laboratoire EXALAB, 33130 Bègles, France; l Laboratoire Bioesterel, 06210 Mandelieu La Napoule, France; m Parasitology-Mycology Laboratory, CH Andrée-Rosemon, 97300 Cayenne, French Guiana; n Department of Biology, Immunology and Parasitology, Cayenne Hospital Center, 97300 Cayenne, French Guiana; o French Military Health Service, French Armed Forces Centre for Epidemiology and Public Health (CESPA), 13002 Marseille, France; p CH Antibes, 06600 Antibes, France

**Keywords:** *Cryptosporidium*, Outbreak, France, Waterborne, Foodborne

## Abstract

*Cryptosporidium* is a known foodborne pathogen, ranked fifth out of 24 among foodborne parasites in terms of importance and a cause of many cryptosporidiosis outbreaks worldwide. In France, very few outbreaks were reported before 2017, and data recently obtained by the Expert Laboratory of the Cryptosporidiosis National Reference Center (CNR-LE-Cryptosporidiosis) have shown that outbreaks are in fact common and frequently underreported. In this work, we aim to report the characteristics of outbreaks detected in France during the period 2017–2020 and present a summary of investigations carried out by the CNR-LE-Cryptosporidiosis. During the study period, there were eleven cryptosporidiosis outbreaks, including three with no identified origin. Among the eight identified outbreaks: six were due to water contamination (five tap water and one recreational water), one was due to direct contact with infected calves, and one was due to consumption of contaminated curd cheese. Among these outbreaks, five of them exceeded one hundred cases. Recent results obtained by the CNR-LE-Cryptosporidiosis revealed the multiannual occurrence of *Cryptosporidium* outbreaks in France. Waterborne outbreaks were more frequently detected, while foodborne outbreaks which are more difficult to detect were likely underreported.

## Highlights

• Massive *Cryptosporidium* outbreaks were detected in France recently.
• Waterborne origin appeared predominant.
• Foodborne origin is probably strongly neglected.
• Develop adapted monitoring and preventing strategies could reduce cryptosporidiosis

## 1 Introduction

*Cryptosporidium* is a known foodborne pathogen, ranked fifth out of 24 among foodborne parasites in terms of importance and a cause of many cryptosporidiosis outbreaks worldwide (World Health Organization, Nations, F. and A.O. of the U, 2014). Between 2011 and 2016, *Cryptosporidium* was responsible for 63% of reported outbreaks due to protozoan waterborne transmission and was reported as the second leading cause of diarrheal disease and death in children in developing countries (Efstratiou et al., 2017; GBD Diarrhoeal Diseases Collaborators, 2017; Ryan et al., 2018). Due to its life cycle, low infective dose, chlorine resistance (Korich et al., 1990; Temesgen et al., 2021), and evasion of water filtration (Wood et al., 2019), *Cryptosporidium* is largely responsible for outbreaks. One of the main reported outbreaks occurred in 1993 in Milwaukee, Wisconsin, United States, and was due to water contamination, leading to more than 400,000 infections in humans and 69 deaths (Lefebvre et al., 2021; MacKenzie et al., 1995). *Cryptosporidium* oocysts are extremely hardy and are easily transmitted through contaminated water sources and fresh produce. Oocysts have been isolated from a variety of foodstuffs, particularly fruits, vegetables and shellfish. Indeed, many foodborne outbreaks were reported mainly from consumption of raw fruit, apple cider, dairy products, and vegetables worldwide (Blackburn et al., 2006; Deng and Cliver, 2000; Millar et al., 2002; Monge and Chinchilla, 1996; Ursini et al., 2020; Vuong et al., 2007). The emergence of *Cryptosporidium* as a leading cause of diarrheal illness worldwide and the potential public health and economic consequences of outbreaks have highlighted the need for rapid, sensitive, and reliable protocols for the detection and differentiation of *Cryptosporidium* spp. in complex matrices (Guo et al., 2021; Millar et al., 2002; O'Leary et al., 2021). The aim of this publication is to provide a summary of reported cryptosporidiosis outbreaks in France between 2017 and 2020. Corresponding epidemiological details are not presented since some specific publications are already published (*Article - Bulletin épidémiologique hebdomadaire [WWW Document*], 2021; *Épidémie de cryptosporidiose signalée dans les Alpes-Maritimes, en France [WWW Document*], 2021) or in press or subject to confidentiality.

## 2 Methods

### 2.1 Surveillance and alerts

The Expert Laboratory of the Cryptosporidiosis National Reference Center (CNR-LE Cryptosporidiosis) is responsible for the active surveillance of the risk of outbreak occurrence in France. Surveillance and alerts include: 1) A weekly prospective surveillance, which is carried out thanks to data analysis provided by the French “Réseau Sentinelles”. The Réseau Sentinelles is a network of volunteer general practitioners and pediatricians, working throughout the metropolitan regions of France. Its goal is to provide clinical surveillance in France for 10 health indicators. Regarding cryptosporidiosis surveillance, the indicator dedicated to the occurrence of acute diarrhea is used. In cases of peaks of acute diarrhea (>250 cases for 100,000 inhabitants), the members of the CNR-LE alert their regional contacts to investigate the associated risk of outbreak; 2) Active surveillance, which is carried out by the 50 public and 15 private laboratories participating in the CNR-LE-Cryptosporidiosis network in France, which alert the CNR-LE-Cryptosporidiosis for further investigations when abnormal clustering of cryptosporidiosis is detected; 3) Alerts, which are given by the CNR-LE-Cryptosporidiosis to the members of its network in the event of a grouped detection of identical *Cryptosporidium* subtypes (based on *gp60* gene sequencing) to proceed to further investigations; and 4) Finally, alerts, which are given by the public health agencies to the CNR-LE-Cryptosporidiosis of a suspected outbreak.

Once identified, each outbreak is notified to the public health agencies (Santé Publique France). Parasitological investigations from both stool samples and matrices suspected to be at the origin of the outbreak are performed by the CNR-LE-Cryptosporidiosis which compares identified subtypes. Epidemiological investigations are performed by the public health agencies (symptom duration, incubation, age, gender, attack rate, epidemiological curves, etc.).

Each outbreak is recorded in the database of the CNR-LE-Cryptosporidiosis and described in the corresponding annual report available online: http://cnrcryptosporidioses.chu-rouen.fr/rapports-annuels/.

### 2.2 Parasitological investigations

The CNR-LE-Cryptosporidiosis is in charge of performing parasitological investigations on samples of both clinical and environmental origins. In cases of suspected outbreaks, the CNR-LE-Cryptosporidiosis performs microscopy analyses and DNA detection on each sample received to confirm or refute the outbreak. Detailed parasitological screening methods have already been published (Costa et al., 2020). Microscopy is based on negative staining method of Heine (Khanna et al., 2014); DNA is extracted and detection of *Cryptosporidium* species is based on real time PCR (Hadfield et al., 2011; Valeix et al., 2020) and GP60 subtyping is subsequently performed (Sulaiman et al., 2005). In cases of confirmed outbreaks, the French national services (Santé Publique France, Agences Régionales de Santé, etc) are in charge of epidemiological investigations.

## 3 Results and discussion

Eleven outbreaks were identified over 4 years from 2017 to 2020. Data are summarized in Table 1. These outbreaks occurred in many regions of the French territory (including overseas) highlighting the ubiquitous distribution of the parasite. Before 2017, only 6 cryptosporidiosis outbreaks in France were reported in the literature (Beaudeau et al., 2008; Dalle et al., 2003; Mosnier et al., 2018). Many reasons could explain the increased number of reported cases of cryptosporidiosis outbreaks in France: i) the proactive monitoring of the CNR-LE-Cryptosporidiosis, ii) the growing number of laboratories in the CNR-LE-Cryptosporidiosis network, iii) the democratisation of syndromic and multiplex molecular methods allowing parasitological investigations which were not initially suspected, and iv) the improvement of food and environmental screening methods (de Roubin et al., 2002; Razakandrainibe et al., 2020; Utaaker et al., 2015).

**Table 1:** Main data of outbreaks in France and overseas departments from 2017 to 2020.

Detection	Region	Number of cases	Setting	Origin	Species	Gp60 subtype	Additional sampling	Evidence^b^
June 2017	Occitanie	100 (estimated) 87 (symptomatic) 13 (laboratory confirmed)	Military community	Tap water	*C. hominis*	IbA10G2	Water (positive to *C. hominis* IbA10G2)	D + G + E
November 2017	Pays de la Loire	180 (symptomatic) 12 (laboratory confirmed)	Community (high school)	Curd cheese	*C. parvum*	IIaA15G2R1	Water (negative) Calves (positive to *C. parvum* IIaA15G2R1)	D + G + E
March 2018	French Guiana	51 (estimated) 16 (laboratory confirmed)	Civilian and military populations	Tap water	*C. hominis*	IbA10G2	Water positive to *C. parvum IIdA19G2*.	D + G + E
August 2018	Grand Est	21 (laboratory confirmed)	Global population	Undefined	*C. hominis*	IaA22R2	NA	G
September 2019	Nouvelle Aquitaine	4 (laboratory confirmed)	Vacationers	Recreational water (lake)	Undefined		Sediment positive to *Cryptosporidium* sp.	G + E
April 2019	Hauts-de-France	267 (symptomatic) 1 (laboratory confirmed)	Global population	Tap water^a^	Undefined		No	D + G
September 2019	Auvergne-Rhône-Alpes	160 (symptomatic) 9 (laboratory confirmed)	Global population	Tap water^a^	Undefined		Water (positive to *Cryptosporidium* sp.)	D + G + E
October 2019	Normandie	12 (laboratory confirmed)	Professional exposure	Direct contamination	*C. parvum*	IIaA15G2R1	Calves (positive to *C. parvum* IIaA15G2R1)	G+ E
November 2019–2020	Provence-Alpes-Côte d'Azur	Several thousands (estimated) 137 (laboratory confirmed)	Global population	Tap water	*C. parvum*	IIdA22G1	Water (positive to *C. parvum*)	D + G + E
2020	Nouvelle Aquitaine	16 (laboratory confirmed)	Global population	Undefined	*C. parvum*	IIdA18G1	No	G
2020	Occitanie	12 (laboratory confirmed)	Global population	Undefined	Not investigated		No	G

a Tap water contamination due to sewage contamination.

b Evidence for association with purposed origin: G = genotyping / D = descriptive and E = environmental investigations.

In November 2017, an outbreak of gastroenteritis occurred in a high school. Cases were defined as students or high school staff presenting at least one of the following symptoms: diarrhea, vomiting, fever or abdominal pain during the period of the outbreak. The public health agencies performed epidemiological investigations. A retrospective cohort study was done using a questionnaire: 180 symptomatic cases were identified and confirmed to *C. parvum* IIaA15G2R1. The drinking water network was investigated and *Cryptosporidium* sp. was not detected. Finally, investigations focused on a meal suspected to be at the origin of the outbreak, more precisely an unpasteurized curd cheese. The exact batch of curd cheese served at the school could not be analysed due to delays in outbreak investigation. However, the same *C. parvum* subtype (IIaA15G2R1) was found in 3 out of 4 sampled calves at the corresponding dairy farm. Hygiene measures were subsequently reinforced at the farm. An article has been written (SPF, 2022; Loury et al., 2019).

In October 2019, a small outbreak occurred in 12 firefighters in charge of a road accident involving a cattle truck (attack rate 100%). The cattle truck was overturned in the ditch and the firefighters had to move the dead animals. They were in direct contact with the feces of animals contaminated with *C. parvum* IIaA15G2R1. Digestive disorders were observed in the firefighters on average 7 days after the intervention. The firefighters were infected with the *C. parvum* IIaA15G2R1 subtype. No article is planned on this outbreak.

In September 2019, a small outbreak occurred in four vacationers accidentally exposed to contaminated recreational water. They presented digestive disorders 7–10 days after swimming in a lake. *Cryptosporidium* subtype from clinical sample could not be obtained. PCR investigation of the sediment of the lake revealed presence of *Cryptosporidium* species. No article is planned on this outbreak.

In April and September 2019, two distinct outbreaks were due to sewage contamination in tap water networks. Respectively 267 and 160 individuals were symptomatic and *Cryptosporidium* was detected thanks to the use of syndromic PCR approaches on 1 and 9 stool samples respectively. Polymicrobial contamination was identified simultaneously (*Campylobacter jejuni)*. No article is planned on these two outbreaks.

In June 2017, an outbreak occurred in military trainees. Cases were defined as patients who had diarrhea or vomiting or abdominal pain in June 2017, and present at the military camp during the 15 days prior to symptom onset. A total of 100 cases were estimated, 87 reported their symptoms (by questionnaire) and 13 were laboratory confirmed. Patients were infected by *C. hominis* IbA10G2. Mean incubation time was about 8 days. Food and water were suspected to be at the origin of the contamination. All investigations performed on food were negative, but investigations on water were positive, revealing contamination of the water network by *C. hominis* IbA10G2. An article is under review by authors before submission.

In 2018, an outbreak occurred in French Guiana. Cases were observed in both civilian and military populations. A “confirmed case” was defined as a patient living or stationed in Maripasoula between January 1st and May 31st 2018, with gastrointestinal illness (diarrhea or nausea or vomiting or abdominal pain), and a *Cryptosporidium*-positive stool test. A “possible case” was defined as a patient living in close contact (same household and time) with a confirmed case, with diarrhea but with no stool examination performed. A “control case” was defined as a subject with the same environmental exposure at the same time but who did not present symptoms. Sixteen cases were laboratory confirmed and revealed contamination by *C. hominis* IbA10G2. A total of 51 cases were estimated. For facility, investigations were performed in the military population. Tap water consumption was the only common risk factor identified. Water sampling performed after the outbreak revealed contamination by *C. parvum* IIdA19G2. An article has been published on this outbreak (Menu et al., 2022).

At the end of 2019, the largest outbreak ever described in France occurred in the Provence-Alpes-Côte d'Azur (PACA) region. Confirmed cases were defined as presenting digestive disorders after the 1st October 2019 and positive to *Cryptosporidium* species. Thus, a total of 137 cases were laboratory confirmed *C. parvum* IIdA22G1. Investigations of water sources revealed contamination with various *C. parvum* subtypes (IIdA22G1 / IIaA15G2R1 / IIdA17G2/ IIdA18G1 / IIaA17G1R1) in a context of an important leaching which occurred during periods of heavy precipitation. Due to the high number of confirmed cases, a cross-sectional survey was carried out in the global population to determine the real incidence of the disease. In this survey, a case was defined as anyone reported to present diarrhea or abdominal pain between October 15th and December 15th, 2019, associated or not with vomiting and other symptoms. The exact number of cases is still under evaluation but is already considered to be several thousands of cases. In terms of management, bottled water was supplied to the exposed population until ultrafiltration and/or ultraviolet were implemented. The economic consequences were high with a cost of several hundreds of thousands of euros. The internal report of the national public health agency “Santé Publique France” is being finalized before international publication.

Origins of contamination could not be identified in three outbreaks. In two of them, outbreaks were suspected late based on retrospective analysis and revealed abnormal clustering of cases with identical subtypes. Questioning of patients did not identify any sufficient correlation between cases to strongly suspect a common origin of contamination and environmental investigations were not done according to public health agency recommendations. In the last case of 2020, the reason was slightly different; an alert was given by a partner laboratory of the CNR-LE-Cryptosporidiosis having retrospectively identified an abnormal clustering of cryptosporidiosis cases. Due to the increased SARS-CoV-2 activity of the corresponding laboratory, analysis of parasitological investigations was done only once yearly (at the end of the year) and stool samples were not available for further investigations when the outbreak was suspected.

Overall, regarding the distribution of species in the eleven reported outbreaks, four outbreaks were due to *C. parvum* and three to *C. hominis*. Regarding gp60 subtypes, *C. parvum* IIaA15G2R1 and *C. hominis* IbA10G2 were predominant, which is coherent with the human distribution of cases in France between 2017 and 2019 (Costa et al., 2020). The anthroponotic *C. hominis* IbA10G2 genotype is known as the worldwide dominant *C. hominis* subtype and is frequently involved in outbreaks implicating waters in England and Wales (Chalmers et al., 2019). The *C. parvum* IIaA15G2R1 subtype is recognized as “hypertransmissible” and frequently identified in both sporadic cases and outbreaks worldwide (Cacciò and Chalmers, 2016; Chalmers et al., 2019; Khan et al., 2018). Among other *C. hominis* subtypes implicated in outbreaks in France, we can cite IaA22R2. Unfortunately, in this case, the origin of the outbreak was not identified. This subtype is poorly documented in the literature: it was described in one sporadic case in the UK after traveling to Pakistan, and in one child in Nigeria (Chalmers et al., 2008; Molloy et al., 2010). The *C. parvum* IIaA15G2R1 subtype was implicated in two of the eleven outbreaks between 2017 and 2020: one due to direct contamination and the other probably due to curd cheese contamination. The common denominator was cattle contamination by the IIaA15G2R1 subtype. The *C. parvum* IIaA15G2R1 subtype is largely reported in both human and livestock cryptosporidiosis cases (Cacciò and Chalmers, 2016; Costa et al., 2020; Feng et al., 2018). A milk contamination with this subtype was already suspected in an outbreak that occurred in 2013 in the Yorkshire region, UK (Chalmers et al., 2019). A similar outbreak occurred in the Pays de la Loire region, France in November 2017, the milk contamination could not be proven but cattle from the corresponding farm were infected by the subtype. *Cryptosporidium* screening from milk is very difficult and no standard method exists. Generally, detection of *Cryptosporidium* in food involves isolating the parasite from the foodstuff and then detecting it using a variety of methods including microscopy, immunofluorescent antibody staining, and DNA-based detection methods. Recently, the International Organization for Standardization (ISO) published guidelines for the detection and enumeration of *Cryptosporidium* and *Giardia* in fresh leafy green vegetables and berry fruits (ISO 18744:2016). Standardized methods for detecting *Cryptosporidium* in/on meat, fish and shellfish are still not available. Regarding the two other identified *C. parvum* subtypes (IIdA22G1 and IIdA18G1) responsible for outbreaks in France, they have already been detected in calves, lamb and goat kids in European countries including France (Björkman et al., 2015; Broglia et al., 2008; Geurden et al., 2007; Mammeri et al., 2019; Plutzer and Karanis, 2007; Taylan-Ozkan et al., 2016). This suggests a contamination of environmental origin.

Overall, results suggest a high implication of water in cryptosporidiosis outbreaks in France. Several hypotheses could explain this: i) water sources could be frequently contaminated by oocysts both of human and animal origin: in France livestock farms are numerous which can generate a strong environmental zoonotic pressure and soil leaching could at least partially explain water contamination; ii) numerous drinking water treatment plants are probably ineffective against parasitological contamination (mainly based on sedimentation and chlorination); iii) in addition, in France, microbiological criteria for drinking water are exclusively based on bacteria detection; iv) a large number of individuals drink tap water: in France, the volume of water consumed per day per inhabitant is estimated at 148 litters (including 1% for drinking water: 1.5 L per day per inhabitant) (https://www.cieau.com/le-metier-de-leau/ressource-en-eau-eau-potable-eaux-usees/quels-sont-les-usages-domestiques-de-leau/); v) water investigations are relatively easy with a standardized method for oocyst screening (NFT90–455) (*NF T90-455 [WWW Document*], 2022). However, in France, few recreational water outbreaks have been reported. In England and Wales, the most common vehicle of *Cryptosporidium* outbreaks was recreational water, especially swimming pools (Chalmers et al., 2019). According to European Centre for Disease prevention and Control reports, Ireland, UK are among the main European reporters. Human cryptosporidiosis became a notifiable disease in Ireland in January 2004 and in UK in 2010. *Cryptosporidium* spp. are notifiable as causative agents of human infection in UK, even if, there is no legal obligation to report outbreaks apart from those that are considered food-borne (Boudou et al., 2021; Chalmers et al., 2019). Considering cryptosporidiosis in France as a notifiable disease together with a stronger implication from public health agencies could greatly improve reporting. For example, in the UK, Health protection teams without exceedance monitoring detected fewer outbreaks than those with exceedance monitoring. In addition, using a system to detect common exposures or always administering a risk-factor questionnaire improved detection of outbreaks (Chalmers et al., 2018).

Foodborne origins of outbreaks are probably also underestimated in France. Since 2016, an international standardardized method exists for the detection and enumeration of *Cryptosporidium* (and *Giardia*) in fresh leafy green vegetables and berry fruits: ISO 18744:2016. However, the recovery of *Cryptosporidium* oocysts varies according to food matrices, sometimes demonstrating weak performances (especially with saponin-rich matrices) (Razakandrainibe et al., 2020; Utaaker et al., 2015). Thus, *Cryptosporidium* screening from food matrices appears very complicated and sensitivity is low. In addition, due to the long incubation period of cryptosporidiosis, food matrices suspected of being contaminated are often no longer available at the time of the investigations.

Nevertheless, this study has some weaknesses. The origin of some outbreaks could not be identified and epidemiological data were few detailed (further developed in subsequent publications or public health agency reports). The data summarized in this article suggest that *Cryptosporidium* outbreaks in France are now more identified than before (11 identified outbreaks between 2017 and 2020 versus previously). A better recognition of cryptosporidiosis from general practitioners, the global population, laboratories and public health agencies could improve outbreak surveillance and detection in France, as already reported in the UK (Chalmers et al., 2018).

## 4 Conclusion

Recent data obtained by the CNR-LE-Cryptosporidiosis revealed the multiannual occurrence of *Cryptosporidium* outbreaks in France. Compared to historically published data, such a high frequency of outbreaks in France was unexpected. This shows good surveillance from the CNR-LE-Cryptosporidiosis and its partners even if some outbreaks probably remain undetected. The development of syndromic approaches on stools from suspected outbreaks contributed to the improvement of parasitological monitoring. Waterborne outbreaks appeared mainly implicated in cryptosporidiosis in France. However, screening of both foodborne and recreational water origins needs to be improved. It has been shown that massive outbreaks can occur in France. All considered, it is expected that these data will contribute to developing effective strategies for monitoring and preventing the risk of cryptosporidiosis outbreaks in France.

## French National Network on Surveillance of Human Cryptosporidiosis (not already included in the list of authors)

Anne Debourgogne, Adela Angoulvant, Julie Bonhomme, Brigitte Degeilh, Cathy Chemla, Cécile Garnaud, Coralie Lollivier, Cécile Angebault, Guillaume Desoubeaux, Emilie Fréalle, Frédéric Grenouillet, Françoise Botterel, Ghania Belkadi, Hélène Yera, Gilles Nevez, Xavier Iriart, Isabelle Accoceberry, Julie Brunet, Marc Thellier, Meja Rabodonirina, Milene Sasso, Charline Miossec, Murielle Nicolas, Nicole Desbois, Philippe Poirier, Sandrine Houze, Céline Nourrisson, Gladys Robert, Florence Robert-Gangneux, Yohann Le Govic, Anne Pauline Bellanger, Franck Labbe, Frédéric Janvier, Céline Damiani, Christine Schuttler, Marie Laure Darde, Luc De Gentile, Marie Pierre Hayette, Olivier Duquesnoy, Pierre Flori, Yvon Sterkers, Jean Philippe Duvert, Jean Philippe Lemoine, Pierre Marty, Edith Mazars, Patrick Bastien Marie-odette Guy, Isabelle Villena, Dominique Aubert, Stéphane Lastere, Estelle Cateau, Claudie Leclair, Alida Minoza, Kévin Brunet, Marie-Elisabeth Bougnoux, Nathalie Kapel, Eric Dannaoui, Alexis Valentin, Laura Courtellemont, Joséphine Dorin, Anne Totet, Laura Verdurme, Nawel Ait Ammar, Alicia Moreno Sabater, Jean Menotti, Pascal Millet, Alexander Pfaf, Emmanuel Dutoit, Renaud Piarroux, Emilie Sitterle, Denis Magne, Samia Hamane, Antoine Berry, Cecile Ramade, Gwenolé Prigent, Marie-Laure Dardé, Stéphane Bonacorsi, Muriel Robert, Sylvain Merieau, Nicolas Argy, Laurence Lachaud, Solène Legal, Jean Philippe Argenson, Christelle Pomares Thomas Gueudet, Denis Blanchet, Denis Lemeteil, Pierre Flori, Josephine Dorin.

## Declaration of Competing Interest

The authors declare that they have no known competing financial interests or personal relationships that could have appeared to influence the work reported in this paper.
